# Plastic Fillings of Metallic Salts

**Published:** 1902-11-15

**Authors:** Ernest Schmitt

**Affiliations:** Prof. Chem., Faculty of Science, Lille, France


### PLASTIC FILLINGS OF METALLIC SALTS.

BY ERNEST SCHMITT, PROF. CHEM., FACULTY OF SCIENCE, LILLE, FRANCE.

The author has experimented with magnesium oxychlorid, which he prepares as follow: 75 grains of recently calcined magnesia are triturated with 2½ fluidrams of a magnesium chlorid solution containing 30 percent of the anhydrous salt of a density of 1.285, at 32° Beaume. After triturating these ingredients for two hours and a half, a very hard resisting cement, with a porcelain appearance, is obtained. A piece of this cement that had been prepared four years previously was suspended in distilled water during August, 1900; it lost one-third of its weight but preserved its form and resistance. The author has prepared zinc oxychlorid in the same way, that is, by triturating 80 grains of calcined zinc oxid and 2½ fluidrams of zinc chlorid of a density of 1.634, at 56° Beaume, but the resulting cement is granular and sets very rapidly. He has also prepared calcium oxychlorid by the method already described. This mixture sets in about half an hour, but does not produce a homogeneous mass. Professor Schmitt then conceived the idea of preparing a mixture of calcium and magnesium oxychlorid. The mixture is carried on in a mortar, and produces a very friable, ball-shaped mass, which hardens in half an hour.—*Cosmos*.

